# Long-term outcomes (2 and 3.5 years post-intervention) of the INFANT early childhood intervention to improve health behaviors and reduce obesity: cluster randomised controlled trial follow-up

**DOI:** 10.1186/s12966-020-00994-9

**Published:** 2020-07-25

**Authors:** Kylie D. Hesketh, Jo Salmon, Sarah A. McNaughton, David Crawford, Gavin Abbott, Adrian J. Cameron, Sandrine Lioret, Lisa Gold, Katherine L. Downing, Karen J. Campbell

**Affiliations:** 1 School of Exercise and Nutrition Sciences, Institute for Physical Activity and Nutrition, Deakin University, Geelong, Australia; 2 Global Obesity Centre (GLOBE), Institute for Health Transformation, School of Health and Social Development, Deakin University, Geelong, Australia; 3 Université de Paris, Research Center in Epidemiology and Biostatistics (CRESS), INSERM, INRA, Paris, France; 4 School of Health and Social Development, Deakin University, Geelong, Australia

**Keywords:** Diet, Physical activity, Screen time, Adiposity, Infancy, Pre-school, Parent

## Abstract

**Background:**

The few health behavior interventions commencing in infancy have shown promising effects. Greater insight into their longer-term benefits is required. This study aimed to assess post-intervention effects of the Melbourne INFANT Program to child age 5y on diet, movement and adiposity.

**Methods:**

Two and 3.5y post-intervention follow-up (2011–13; analyses completed 2019) of participants retained in the Melbourne INFANT Program at its conclusion (child age ~ 19 m; 2008–10) was conducted. The Melbourne INFANT Program is a 15-month, six session program delivered within first-time parent groups in Melbourne, Australia, between child age 4-19 m. It involves strategies to help parents promote healthy diet, physical activity and reduced sedentary behavior in their infants. No intervention was delivered during the follow-up period reported in this paper. At all time points height, weight and waist circumference were measured by researchers, children wore Actigraph and activPAL accelerometers for 8-days, mothers reported children’s television viewing and use of health services. Children’s dietary intake was reported by mothers in three unscheduled telephone-administered 24-h recalls.

**Results:**

Of those retained at program conclusion (child age 18 m, *n* = 480; 89%), 361 families (75% retention) participated in the first follow-up (2y post-intervention; age 3.6y) and 337 (70% retention) in the second follow-up (3.5y post-intervention; age 5y). At 3.6y children in the intervention group had higher fruit (adjusted mean difference [MD] = 25.34 g; CI_95_:1.68,48.99), vegetable (MD = 19.41; CI_95_:3.15,35.67) and water intake (MD = 113.33; CI_95_:40.42,186.25), than controls. At 5y they consumed less non-core drinks (MD = -27.60; CI_95_:-54.58,-0.62). Sweet snack intake was lower for intervention children at both 3.6y (MD = -5.70; CI_95_:-9.75,-1.65) and 5y (MD = -6.84; CI_95_:-12.47,-1.21). Intervention group children viewed approximately 10 min/day less television than controls at both follow-ups, although the confidence intervals spanned zero (MD = -9.63; CI_95_:-30.79,11.53; MD = -11.34; CI_95_:-25.02,2.34, respectively). There was no evidence for effect on zBMI, waist circumference z-score or physical activity.

**Conclusions:**

The impact of this low-dose intervention delivered during infancy was still evident up to school commencement age for several targeted health behaviors but not adiposity. Some of these effects were only observed after the conclusion of the intervention, demonstrating the importance of long-term follow-up of interventions delivered during early childhood.

**Trial registration:**

ISRCTN Register ISRCTN81847050, registered 7th November 2007.

## Background

Overweight and obesity are prevalent from a young age [1], as are the health behaviours that predispose children to such conditions including poor diet [2, 3], insufficient physical activity [4] and excessive sedentary behavior [5]. The World Health Organisation has highlighted the importance of early intervention [6]. However, few programs have capitalised on the opportunity afforded by very early intervention (prior to 2y of age) to promote health behaviors from their inception [7]. Fortunately this has begun to change with a small number of trials [8–11], including our own [12], beginning to target this key developmental period. Results are promising, with indications of impact on health behaviors [8–12] and weight [9, 13] in the short-term.

A key gap in childhood obesity prevention evidence continues to be lack of longer-term follow-up [7, 14]. This is where early intervention studies stand out. While few in number, the majority include follow-up beyond conclusion of the intervention [15–17]. These interventions, commencing in infancy, found effects on health behaviors and weight were generally not sustained to 5y of age [15, 16] or demonstrated a reduction in the magnitude of effect over time [17]. The small number of studies and heterogeneity in intervention approach leaves much scope for further investigation.

The Melbourne Infant Feeding, Activity and Nutrition Trial (INFANT) Program [12] was amongst this first suite of early childhood interventions aiming to improve health behaviors from infancy and reduce obesity. Using an anticipatory guidance approach [18] and uniquely nesting within existing social groups, relative to usual practice this low dose intervention (6 × 2-h sessions over 15 m) was effective in reducing sweet snack consumption by 25% and television viewing by 25% in children at 20 m of age [12], improving diet quality [2, 19], and in increasing water and vegetable intake amongst children of younger or less educated mothers [20]. It also improved diet quality and feeding practices amongst mothers participating in the intervention [21, 22] but had no impact on fathers’ health behaviors [23] or on child physical activity or zBMI [12].

This paper aims to assess the longer-term intervention effects on child health behaviors and adiposity, and cost of the Melbourne INFANT Program at two and 3.5y post-intervention (child age 3.6y and 5y, respectively).

## Methods

### Study design

Efficacy of the Melbourne INFANT Program (2008–2010; ISRCTN81847050) was assessed mid- and post-intervention using a cluster-randomised controlled trial design and these results have been reported elsewhere [12, 19–21, 23]. Participants were followed-up two (2011–2012) and 3.5 (2013) years after conclusion of the intervention with these post-intervention follow-ups being the focus of this paper, the protocol for which has been published [24]. The CONSORT 2010 statement extension for cluster randomised trials is used in the reporting of this study [25].

### Sample

Recruitment for the initial trial has been previously reported [12, 24]. Briefly, 14 local government areas (LGAs) were randomly selected from those within a 60 km radius of the study centre in Melbourne, Australia. From these, 50% of all eligible maternal and child health centres (a free universal service available to all parents in Victoria, Australia) running first time parent groups within each LGA were randomised to the intervention arm. If either an LGA or centre/group declined, they were replaced with the next on the randomly ordered list. Eligible groups included a minimum of eight consenting parents which was reduced to a minimum of six in disadvantaged areas.

For the follow-up phase, all intervention and control families who remained enrolled in the program at the end of the intervention (*n* = 480; 88.6% of those originally recruited) were recontacted and invited to participate. Renewed written consent was required for participation. Ethics approval for the initial trial and the follow-up (as an extension) were received from Deakin University’s Human Research Ethics Committee (EC 175–2007).

### Intervention

The Melbourne INFANT Program [12] was a 15-month health behavior intervention delivered to first-time parents of infants from approximately 4 m of age. The six 2-h group sessions (occurring approximately once every 3 months) were delivered by dietitians within existing first-time parent groups. The intervention was informed by social cognitive theory and utilised an anticipatory guidance framework [18] to facilitate parents’ acquisition of knowledge and strategies to promote healthy dietary intake, physical activity and sedentary behaviors in line with their infants’ developmental phases over the next 15 months. It was structured around key messages [26] which were reiterated throughout the program and provided a framework for parents to understand how these same messages could be applied at different developmental phases. Such intervention strategies could potentially facilitate longer term maintenance, however, no explicit discussion of the preschool developmental phase was incorporated into the program. During the follow-up phase no further intervention occurred.

The control group received six newsletters delivered approximately once every 3 months over the same period as the intervention. Newsletter topics were unrelated to any of the behaviors under investigation e.g. literacy, common childhood illnesses. Control group participants may have received information on topics related to the intervention from their maternal and child health nurse or other health professional during usual care but this was not assessed.

### Measures

At each follow-up, parents were provided with a questionnaire for self-completion prior to their home visit. This covered demographic information, screen time, health care usage and a number of tertiary outcomes not reported here. At a prearranged time, researchers visited each participant’s home to collect child anthropometric data, fit accelerometers, and collect questionnaires. Dietary recalls were conducted at unscheduled times following the home visit. Baseline trial data collected when infants were 4 m of age (parent reported demographics and researcher measured infant length and weight) were included as covariates in analyses.

#### Anthropometry

Height was measured to the nearest 0.1 cm using a portable stadiometer (Seca 220/217, Hamburg). Weight was measured in light clothing to the nearest 10 g using digital scales (Tanita BWB-800/InnerScan 50, Illinois). Waist circumference was measured to the nearest 0.1 cm using a steel non-stretch tape (Lufkin Executive Thinline W606PM, Maryland). All measures were taken twice by trained research staff, with a third taken if the difference exceeded 0.5 cm/0.5 kg. The mean of the closest two measures was used in analyses. Body mass index (BMI; kg/m^2^) and BMI z-scores (zBMI) were calculated based on World Health Organisation BMI-for-age growth charts [27].

#### Dietary intake

Using the 24-h recall method [28], trained researchers conducted unscheduled telephone interviews with parents on three non-consecutive days, including one weekend day, to capture all food and drink consumed by the child on the previous day. Food measurement booklets were provided to assist parents with estimating quantities. Data were categorised according to the Australian Food, Supplement & Nutrient Database (AUSNUT2007) (Food Standards Australia New Zealand, Canberra, Australia, 2008), with additional infant-specific products added. Coding of all food items was checked for accuracy and completeness by a dietitian. Average daily intakes of fruits (excluding juice), vegetables (excluding potatoes), noncore sweet foods (eg, chocolate, cakes), noncore savory foods (eg, crisps, savory biscuits), noncore drinks (ie, fruit juice, soft drinks), and water were calculated. Variety of fruits was calculated by summing the number of specific fruits reported by parents across all recalls. Juice was excluded. Where multi-variety foods were reported (e.g. fruit salad) a score of 2 or 3, rather than 1, was assigned for the purposes of calculation. The same process was used to calculate vegetable variety. Potato was excluded.

#### Physical activity

Children wore ActiGraph™ accelerometers (Model GT1M, Pensacola) on an elasticised belt at the right hip during waking hours for 8 consecutive days. Movement counts were recorded in 15-s epochs. Light-intensity physical activity (LPA) was defined as > 25 and < 420 counts [29]. Moderate- to vigorous-intensity physical activity (MVPA) was defined as ≥420 counts [29]. Non-wear time, defined as ≥20 min of consecutive zero counts, was removed. Children with at least 4 days of ≥7.4 h of recorded data were included in analyses, which has been shown to provide a reliable estimate of habitual physical activity [30].

#### Sedentary behaviors

Parents reported the time (hours and minutes) their child usually spent watching television/DVDs on a typical weekday and typical weekend day, using items with established reliability [31]. Average daily minutes of television viewing was generated ((weekdays × 5 + weekend × 2)/7). To assess time spent sitting, children wore activPAL™ monitors (PAL Technologies Ltd., Glasgow) on an elasticised belt on the front of the right thigh, midway between the knee and hip, for 8 consecutive days during waking hours. Non-wear time, defined as ≥20 min of consecutive zero counts based on the vertical axis of the accelerometer in the activPAL™, was removed. Children with at least 2 days of ≥6 h of recorded data were included in analyses. Two days of data were selected to maximise inclusion and were shown to be not substantially different from 3+ days of data in sensitivity analyses.

### Economic analysis

Economic evaluation considered the incremental costs of the intervention from a healthcare perspective (costs accrued in the intervention compared to the control arm) and net of any reduction in service use over the period of follow-up. The pre-specified definition of cost-effectiveness assessed costs only in terms of additional costs per unit change in BMI [24]. Intervention costs were calculated at the end of the intervention period [12], and converted to 2018 figures. Over the period of follow-up, parents reported any services used due to concerns over their own or their child’s weight, diet or physical activity. Parents were first asked to indicate whether they had accessed any services for these purposes since last completing a survey. If they responded ‘yes’, they were asked to report the average number of visits, average total cost and out of pocket cost for visits to each of eight commonly utilised health services (e.g. GP, mother-baby or parenting centre) plus any other health professionals they may have visited. Reported service use was valued in 2018 Australian dollars using existing national estimates.

### Data analyses

Participants were analysed on an intention-to-treat basis. Effects of the intervention were assessed at each time point by testing for differences between trial arms on the outcome variables using maximum likelihood linear mixed models with random intercepts for parent groups, to account for clustering. Due to skewness among outcome variables, bootstrapping with 2000 resamples was used to calculate model standard errors and normal-approximation confidence intervals were constructed. Unadjusted models were tested for all outcomes with the exception of the BMI z-score outcomes where baseline values were available and included as covariates. Behavioral outcomes were not available at baseline given children were 4 m old. All physical activity models (unadjusted and adjusted) included average accelerometer wear time. Adjusted models included child age, child sex, and maternal education as covariates. Additionally, mothers’ pre-pregnancy BMI (reported at baseline) was included in models for the BMI and waist circumference z-score outcomes, and child overall energy intake was included in the dietary outcomes models. Since the study outcomes were measured in a variety of units, standardised effect sizes (d) were calculated for the forest plots by dividing adjusted mean differences between treatment groups by the standard deviation of the outcome in the control group. An effect size of d = 1 would indicate the intervention caused an estimated increase of one standard deviation in the outcome, relative to the level of the outcome without intervention. Stata/SE 15.0 (StataCorp, Texas) was used for analyses.

## Results

**Fig. 1:**
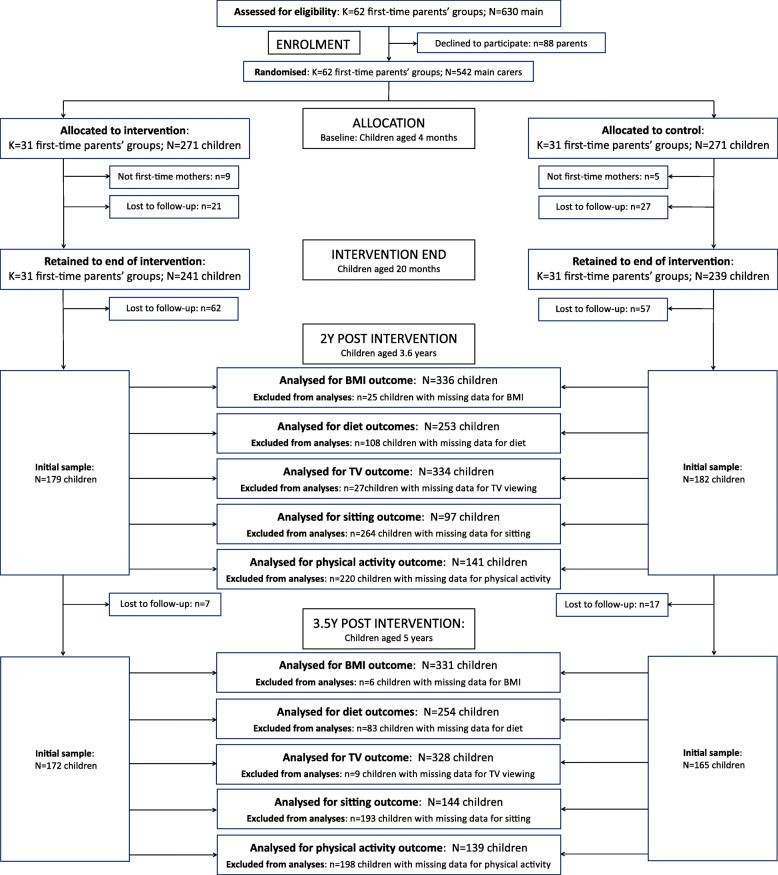
Retention of participants in the Melbourne InFANT Program 2y and 3.5y post intervention. Abbreviations: CI = confidence interval; zBMI = body mass index z-score; PA = Physical activity. Standardised effect sizes (d) greater than 0 indicate estimated outcome values were higher for the intervention group compared to the control group

Participant retention is shown in Fig. 1. Of the 480 families still enrolled at intervention conclusion, 361 (75%) participated in the first follow-up when children were aged approximately 3.6y and 337 (70%) participated in the second follow-up when children were approximately 5.1y. There was no difference in retention between intervention and control conditions. Comparison of baseline characteristics between those retained and lost to follow-up at both time points showed that children retained were younger at baseline, and more likely to have a university educated mother. The retained sample also had higher fruit intake, and lower water intake and television viewing time at the end of the intervention compared to those lost to follow-up. Characteristics of the retained sample are described in Table 1.

**Table 1:** Characteristics of participants retained in follow-up phase

Characteristic	Total sample (*n* = 361)	Control (*n* = 182)	Intervention (*n* = 179)
	*M (SD)*	*M (SD)*	*M (SD)*
Child sex, female (%)	46.0	44.0	48.0
Child age at first follow-up (y)	3.6 (0.2)	3.6 (0.2)	3.6 (0.2)
Child age at second follow-up (y)	5.1 (0.1)	5.0 (0.1)	5.1 (0.1)
Birthweight (g)	3362.0 (595.6)	3368.2 (622.3)	3355.8 (569.3)
Main carer^a^ age at first follow-up (y)	35.7 (4.3)	35.6 (4.3)	35.8 (4.3)
Main carer highest level of education at baseline (child age 4 m) (%)			
Some high school	18.3	17.0	19.6
Completed high school / trade / certificate	21.6	18.7	24.6
University	60.1	64.3	55.9
Main carer employed at first follow-up (%)	50.8	52.4	49.3
Main carer born in Australia (%)	80.3	79.7	81.0
English main language spoken at home (%)	93.9	93.4	94.4

^a^All main carers were mothers apart from one father

**Fig. 2:**
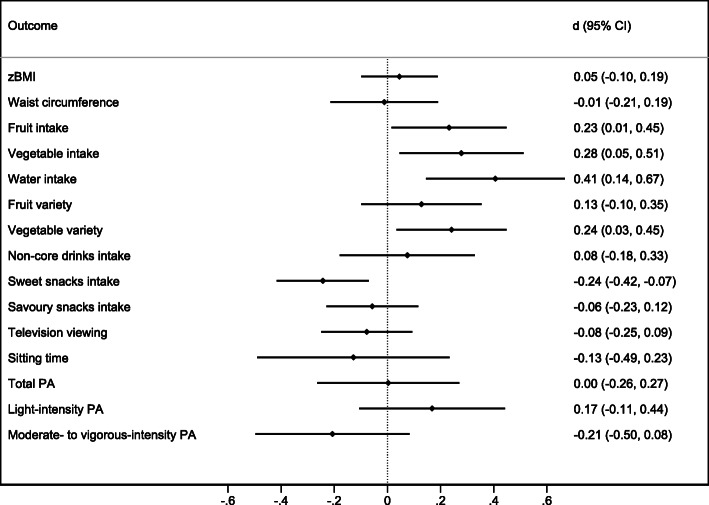
Post-intervention effects at first follow-up (child age 3.6y). Abbreviations: CI = confidence interval; zBMI = body mass index z-score; PA = Physical activity. Standardised effect sizes (d) greater than 0 indicate estimated outcome values were higher for the intervention group compared to the control group

**Fig. 3:**
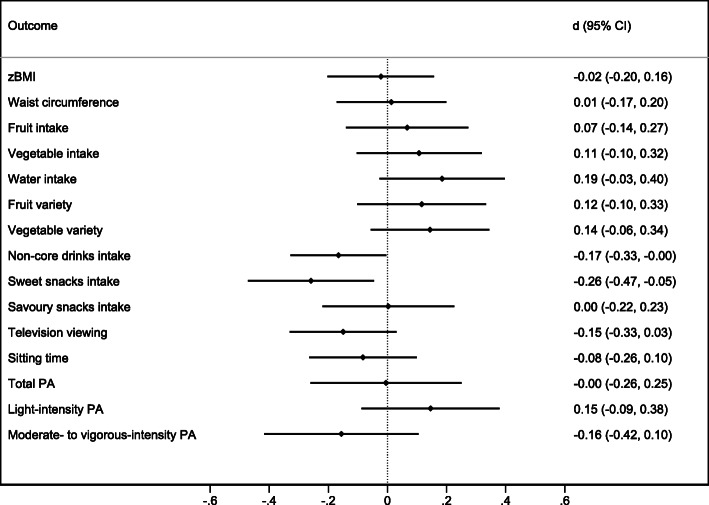
Post intervention effects at second follow-up (child age 5y). Abbreviations: CI = confidence interval; zBMI = body mass index z-score; PA = Physical activity. Standardised effect sizes (d) greater than 0 indicate estimated outcome values were higher for the intervention group compared to the control group

Figures 2 and 3 (and eTable 1) show comparison of the outcomes between intervention and control groups at each follow up assessment. Adjusting for covariates, there was no evidence of an effect of the intervention on adiposity, with the point estimate for waist circumference z-scores falling on the zero line at both follow-ups and for BMI z-score at the second follow-up. At the first follow-up, children who had received the intervention had more favourable dietary intakes across all outcomes than their peers in the control condition. The strongest impacts were seen for fruit (standardised effect size [d] = 0.23, CI_95_ = 0.01,0.45), vegetable (d = 0.28, CI_95_ = 0.05,0.51), water (d = 0.41, CI_95_ = 0.14,0.67), sweet snack intake (d = − 0.24, CI_95_ = -0.42,-0.07) and vegetable variety (d = 0.24, CI_95_ = 0.03,0.45). The magnitude of effects was diminished at the second follow-up with the exception of non-core drinks (d = − 0.17, CI_95_ = -0.33,-0.00) and sweet snack intake (d = − 0.26, CI_95_ = -0.47,-0.05); there also remained weak evidence of a difference for water intake (d = 0.19, CI_95_ = -0.03,0.40). Non-core drinks intake, which showed little between group difference at the first follow-up (d = 0.08, CI_95_ = -0.18,0.33), was lower in the intervention group at the second follow-up (d = − 0.17, CI_95_ = -0.33,-0.00).

The intervention demonstrated no evidence of impact on children’s overall physical activity, with the point estimates falling on the zero line. Similarly for sitting time which, while in the desired direction, had large confidence intervals particularly at the first follow up. While the 95% confidence intervals included zero, television viewing was reported to be lower in the intervention compared to the control group by approximately 10 min/day at the first follow-up (d = − 0.08, CI_95_ = -0.25, 0.09) and 11 min/day at the second follow-up (d = − 0.15, CI_95_ = 0.-0.33,0.03).

Intervention costs were reported previously [12]. In 2018 values, these costs equate to approximately A$633 per family for providing the intervention. Parent-reported service use (for child- or own-weight, diet or activity concerns) over the period of follow-up was similar for intervention and control families (eTable 2), thus there was no evidence of a net saving over time in service use costs to balance against the upfront intervention costs. A minority of parents across both groups reported accessing health care or other services for these purposes: 28% of parents at the first follow-up (3.6y) and 13–14% at the second follow-up (5y). The type of services used were similar between groups at both time points. General Practitioners were the most commonly used service across time, with Maternal and Child Health telephone helplines and paediatricians used more at the 3.6y than 5y follow-up (eTable 2). The services used by families were often provided at fully-subsidised rates (i.e., zero out-of-pocket costs), with average out-of-pocket costs to families only $11–36 per family.

## Discussion

At 3.6y and 5y of age, children whose parent participated in the Melbourne INFANT Program, a low dose intervention aiming to improve early childhood obesity preventive behaviors across the first 18 months of life, had more favourable diet and lower television viewing than their peers, but similar adiposity and physical activity outcomes. Stronger dietary effects were seen 2y post-intervention than at 3.5y post-intervention. The effect sizes observed translate to important impacts at a population level.

At completion of the intervention a similar pattern of more favourable dietary intakes in the intervention compared to the control group was observed, although the magnitude of differences was considerably lower with only sweet snacks intake demonstrating a between-group mean difference where confidence intervals did not cross zero [12]. This suggests that the dietary effects of the intervention became stronger as children got older. This could have been because the intervention messages and strategies taught become more effective with practice or that they may have greater relevance for parents as children become older and their diet typically expands to include more discretionary foods [32].

The findings of this study are in contrast to other very early childhood interventions which have reported impacts being no longer apparent [15, 16] or diminished [17] over the longer term. It is possible that use of existing social groups for delivery of the Melbourne INFANT Program, a unique aspect of this program, may have contributed to this longer term success. Parents participating in the intervention reported discussing program messages with each other between sessions (unpublished data) and we hypothesise that this may have continued after completion of the program. Outside of this study these Maternal and Child Health nurse initiated groups that are universally offered in Victoria run formally with the nurse for 6–8 weeks during the first ~ 3 m of a child’s life, Yet most continue to interact regularly to ~ 18 m of age and beyond, and the vast majority of mothers continue friendships with other group members [33]. It is also possible that parents referred back to the intervention materials as their children reached new developmental stages to help them deal with the arising challenges. The practical strategies taught within the program, and the consistent messages that were modified for each developmental stage, may have equipped parents with ‘tools’ and awareness of the malleability of strategies that they could use and adapt as their children continued to develop, applying these to the new challenges they faced into the preschool years.

Only sweet snacks intake showed a strong sustained effect. Children in the intervention group consumed approximately 20% less sweet snacks than children in the control group. This effect, maintained to 5y of age, was only slightly lower than the 25% difference seen at the end of the intervention. The impact of the intervention on consumption of sweet snacks could have important public health implications given Australian 2-3y olds consume more than 30% of their energy from discretionary foods and the corresponding figure for 4-8y olds is more than 37%, of which sweet snacks is the main contributor [34]. It is interesting to note that mothers’ sweet snack intake was also positively impacted by the intervention [21] suggesting role modelling and restricted availability within the home could help explain these sustained differences.

While there was variability in the strength of effects for dietary outcomes across the two time points, children in the intervention group generally showed more favourable dietary intakes at both follow-ups. The variability may reflect variations in individual child intake across the early childhood period, or may stem from random variation, measurement error, or statistical issues associated with relatively small differences or the size of the sample. Nonetheless clear patterns were observed which may assist in understanding where interventions of this kind are likely to have impact and help in determining required sample sizes to clearly detect such effects. For example, an impact on beverage intake was relatively consistent across time points. Higher water intake was reported for children in the intervention group at intervention conclusion and both follow-ups, and lower non-core drink consumption at intervention conclusion and 5y follow-up. These data suggest that a focus on fluid consumption may be a simple and sustainable target for dietary interventions seeking to reduce total energy intakes and improve overall diet quality [35], as well as targeting other important public health issues such as caries prevention [36]. Given adolescents are the highest consumers of sugar sweetened beverages in the population [34], targeting beverage intake early in life to change this trajectory may be a particularly promising public health strategy.

Children in the intervention group continued to watch less television on average than their control counterparts throughout follow-up. While confidence intervals crossed zero, this average of 11 min lower television viewing per day at 5y represents more than an hour less viewing across the week. Such a reduction is likely to be important for shifting the population curve given only 27% of Australian pre-schoolers meet national guidelines of no more than 1 hour of screen time per day [37]. The differential loss to follow-up of children with higher television viewing at intervention conclusion may have contributed to the reduced capacity to detect differences at follow-up. Across the early childhood period, children progressively move from a strongly parent controlled environment to an environment with greater exposure to competing forces as well as increased child autonomy. It is important that we consider how best to support continuation of healthy behaviors, developed in the early years, across life.

While differences in adiposity, physical activity and sitting were not found, the cumulative impact of multiple improved health behaviors over time and the potential for changed health trajectories means impacts may only become evident over the longer term. Further, given health behaviors have a significant impact on health and wellbeing independent of weight status [36, 38], the positive impact on these behaviors is meaningful even in the light of no change in adiposity.

Limitations of the current study include loss to follow-up, with higher attrition amongst families with less educated mothers, and differential behavioral profiles for fruit intake, water and television viewing at intervention conclusion for those retained and lost to follow-up. Given the intervention showed greater impact for children of less educated mothers at its conclusion [20], this differential attrition may have resulted in an underestimate of effect sizes. Similarly, more favourable behavior profiles (higher fruit and lower television) in the retained sample allowed less opportunity to demonstrate improvement at follow-up. It also reflects the difficulty of retaining those most in need of support. This highlights the need for translation beyond the research setting of programs shown to be effective, as hard to reach populations are more likely to engage with community delivered programs than with research programs [14]. While the retained sample at both follow ups approximated the sample sizes that power calculations were based on [24], the low numbers of children with complete physical activity and dietary data at follow-up may also have impacted the power to detect differences. Further, the minimum detectable differences the study was powered to detect may not be the minimum important differences, given the study was constrained by the predefined sample size. For example, the study was powered to detect a 20 min reduction in sedentary time. While a much smaller reduction might be considered important and meaningful at a population level, a much larger sample would be required to detect a smaller difference with 95% confidence. Finally, given the number of statistical comparisons undertaken, there is a risk of Type 1 error. Strengths of the study included high retention, the gold standard population-based outcome measures employed, and the repeated measures.

## Conclusions

It is very promising that this low dose intervention continued to show impact up to 3.5y post intervention on some of the health behaviors targeted. Assessing the value of an early childhood intervention based solely on impacts immediately post intervention may overlook longer term benefits which are potentially more sustainable. Results of this study further highlight the importance of incorporating longer term follow-up into intervention studies, regardless of the initial impact of the intervention. This may be particularly important for the early childhood period where behaviors are undergoing rapid development and trajectories for longer term health behaviors are being established.

## Supplementary information

**Additional file 1: eTable 1.** Post-intervention effects at first (child age 3.6y) and second (child age 5y) follow-up. **eTable 2.** Parent-reported service use and costs at follow-up (Costs in 2018 Australian dollars)

## Data Availability

The datasets generated and/or analysed during the current study are not publicly available due to ethical restrictions related to the consent given by participants at the time but are available from the corresponding author on reasonable request and pending approval from the relevant ethics committees.

## Abbreviations

BMI: Body mass index
LGA: Local government area
CI: Confidence interval
