# Supplementary material for: Long-term outcomes (2 and 3.5 years post-intervention) of the INFANT early childhood intervention to improve health behaviors and reduce obesity: cluster randomised controlled trial follow-up
Source: Int J Behav Nutr Phys Act. 2020 Jul 25;17:95. doi: 10.1186/s12966-020-00994-9 (PMC7382091; doi:10.1186/s12966-020-00994-9)
Supplement: Supplementary file 1 — Additional file 1: eTable 1. Post-intervention effects at first (child age 3.6y) and second (child age 5y) follow-up. eTable 2. Parent-reported service use and costs at follow-up (Costs in 2018 Australian dollars) [file 12966_2020_994_MOESM1_ESM.docx]

**eTable 1: Post-intervention effects at first (child age 3.6y) and second (child age 5y) follow-up**

|  | **Distribution of the outcomes** | | | | **Effects of the intervention** | | **Effects of the intervention accounting for covariates** | |
| --- | --- | --- | --- | --- | --- | --- | --- | --- |
|  | **Control** | | **Intervention** | |  |  |  |  |
|  | **Mean (SD)** | **Median (IQR^a^)** | **Mean (SD)** | **Median (IQR^a^)** | **Mean difference^b^**  **(95% CI)** | **p** | **Mean difference^c^**  **(95% CI)** | **p** |
| **First post-intervention follow-up** | |  |  |  |  |  |  |  |
| zBMI | 0.6 (0.9) | 0.7 (0.0, 1.2) | 0.7 (0.9) | 0.7 (0.2, 1.2) | 0.05 (-0.08, 0.19) | .451 | 0.03 (-0.10, 0.15) | .658 |
| Waist circumference z-score | 0.5 (1.1) | 0.5 (-0.2, 1.2) | 0.5 (1.1) | 0.6 (0.0, 1.1) | 0.02 (-0.21, 0.24) | .893 | -0.01 (-0.24, 0.21) | .908 |
|  |  |  |  |  |  |  |  |  |
| Fruit intake (g/d) | 190.8 (109.3) | 167.3 (108.5, 268.1) | 216.6 (121.3) | 198.2 (138.9, 269.0) | 25.80 (1.37, 50.23) | .038 | 25.34 (1.68, 48.99) | .036 |
| Vegetable intake (g/d) | 80.4 (69.7) | 69.0 (30.1, 108.9) | 96.9 (73.7) | 77.8 (48.0, 125.9) | 17.22 (-0.20, 34.63) | .053 | 19.41 (3.15, 35.67) | .019 |
| Water intake (g/d) | 475.6 (279.3) | 433.3 (283.3, 583.3) | 586.9 (360.3) | 529.2 (333.3, 750.0) | 111.32 (48.33, 174.31) | .001 | 113.33 (40.42, 186.25) | .002 |
| Fruit variety score | 2.6 (1.4) | 2.3 (1.7, 3.3) | 2.7 (1.4) | 2.7 (1.7, 3.3) | 0.14 (-0.20, 0.47) | .419 | 0.18 (-0.14, 0.50) | .269 |
| Vegetable variety score | 2.1 (1.6) | 2.0 (1.0, 3.3) | 2.5 (1.4) | 2.3 (1.7, 3.3) | 0.35 (0.04, 0.67) | .027 | 0.38 (0.05, 0.71) | .023 |
| Non-core drinks intake (g/d) | 80.5 (119.9) | 31.5 (0.0, 125.0) | 90.7 (163.2) | 13.9 (0.0, 113.8) | 10.21 (-18.38, 38.80) | .484 | 8.94 (-21.63, 39.50) | .567 |
| Sweet snacks intake (g/d) | 28.6 (23.4) | 25.7 (10.1, 40.4) | 22.8 (18.6) | 19.3 (8.0, 33.7) | -5.84 (-10.07, -1.61) | .007 | -5.70 (-9.75, -1.65) | .006 |
| Savoury snacks intake (g/d) | 9.6 (15.4) | 4.3 (0.0, 12.6) | 9.0 (10.9) | 5.5 (0.0, 14.0) | -0.64 (-3.14, 1.86) | .618 | -0.87 (-3.52, 1.78) | .519 |
|  |  |  |  |  |  |  |  |  |
| Television viewing (min/d) | 118.0 (123.9) | 90.0 (60.0, 120.0) | 113.2 (116.5) | 91.1 (60.0, 120.0) | -4.81 (-26.45, 16.83) | .663 | -9.63 (-30.79, 11.53) | .372 |
| Sitting time (min/day) | 317.3 (81.7) | 313.1 (262.6, 365.9) | 309.9 (82.4) | 304.7 (258.2, 344.4) | -7.33 (-34.60, 19.95) | .599 | -10.47 (-40.04, 19.10) | .488 |
| Total physical activity: LMVPA (mins/d) | 316.1 (42.6) | 319.0 (284.0, 335.9) | 318.0 (43.0) | 318.5 (290.7, 346.3) | 1.90 (-10.19, 14.00) | .758 | 0.15 (-11.23, 11.52) | .980 |
| LPA (mins/d) | 243.1 (29.4) | 238.9 (220.0, 260.5) | 249.2 (30.9) | 248.8 (231.6, 269.6) | 6.12 (-2.69, 14.92) | .173 | 4.94 (-3.13, 13.02) | .230 |
| MVPA (mins/d) | 73.0 (23.2) | 70.0 (56.3, 85.3) | 68.8 (23.1) | 64.0 (53.5, 82.6) | -4.22 (-10.43, 2.00) | .184 | -4.80 (-11.52, 1.92) | .162 |

|  | **Distribution of the outcomes** | | | | **Effects of the intervention** | | **Effects of the intervention accounting for covariates** | |
| --- | --- | --- | --- | --- | --- | --- | --- | --- |
|  | **Control** | | **Intervention** | |  |  |  |  |
|  | **Mean (SD)** | **Median (IQR^a^)** | **Mean (SD)** | **Median (IQR^a^)** | **Mean difference^b^**  **(95% CI)** | **p** | **Mean difference^c^**  **(95% CI)** | **p** |
|  |  |  |  |  |  |  |  |  |
| **Second post-intervention follow-up** | |  |  |  |  |  |  |  |
| zBMI | 0.5 (0.9) | 0.6 (0.0, 1.1) | 0.6 (1.0) | 0.5 (0.0, 1.2) | -0.03 (-0.21, 0.15) | .724 | -0.02 (-0.19, 0.15) | .808 |
| Waist circumference z-score | 0.6 (1.1) | 0.6 (0.0, 1.2) | 0.6 (1.2) | 0.7 (-0.1, 1.3) | 0.00 (-0.27, 0.27) | .985 | 0.01 (-0.18, 0.21) | .887 |
|  |  |  |  |  |  |  |  |  |
| Fruit intake (g/d) | 210.4 (121.9) | 179.8 (125.5, 294.0) | 210.6 (114.6) | 198.6 (135.2, 272.7) | 2.31 (-25.77, 30.39) | .872 | 8.16 (-17.07, 33.40) | .526 |
| Vegetable intake (g/d) | 116.1 (90.6) | 86.7 (46.1, 163.0) | 122.5 (79.6) | 113.5 (67.3, 155.2) | 6.97 (-12.04, 25.98) | .472 | 9.71 (-9.43, 28.86) | .320 |
| Fruit variety score | 2.5 (1.3) | 2.3 (1.3, 3.3) | 2.6 (1.2) | 2.3 (2.0, 3.3) | 0.10 (-0.18, 0.37) | .493 | 0.15 (-0.13, 0.42) | .297 |
| Vegetable variety score | 2.2 (1.5) | 2.0 (1.0, 3.3) | 2.3 (1.5) | 2.0 (1.3, 3.3) | 0.11 (-0.20, 0.42) | .477 | 0.22 (-0.09, 0.53) | .160 |
| Water intake (g/d) | 544.0 (318.2) | 500.0 (333.3, 716.7) | 596.6 (338.5) | 513.3 (350.0, 802.7) | 52.59 (-16.28, 121.46) | .134 | 58.92 (-8.56, 126.39) | .087 |
| Non-core drinks intake (g/d) | 96.7 (166.4) | 35.5 (0.0, 126.0) | 66.1 (113.2) | 3.5 (0.0, 87.0) | -30.51 (-57.45, -3.57) | .026 | -27.60 (-54.58, -0.62) | .045 |
| Sweet snacks intake (g/d) | 34.1 (26.4) | 30.0 (14.1, 48.2) | 27.4 (25.1) | 22.9 (8.3, 41.7) | -6.62 (-12.25, -0.98) | .021 | -6.84 (-12.47, -1.21) | .017 |
| Savoury snacks intake (g/d) | 13.6 (16.1) | 8.7 (2.0, 19.4) | 14.0 (15.0) | 9.6 (1.4, 21.1) | 0.40 (-4.10, 4.90) | .862 | 0.05 (-3.54, 3.63) | .980 |
|  |  |  |  |  |  |  |  |  |
| Television viewing (min/d) | 102.7 (75.5) | 85.7 (60.0, 120.0) | 93.1 (65.2) | 77.1 (60.0, 120.0) | -10.14 (-23.58, 3.29) | .139 | -11.34 (-25.02, 2.34) | .104 |
| Sitting time (min/day) | 541.5 (213.1) | 518.2 (371.6, 717.1) | 550.0 (206.8) | 517.1 (391.5, 678.6) | -14.05 (-83.75, 55.64) | .693 | -20.67 (-58.85, 17.51) | .289 |
| Total physical activity: LMVPA (mins/d) | 326.6 (49.9) | 318.0 (294.0, 361.6) | 333.1 (47.3) | 330.0 (294.1, 373.6) | 6.45 (-4.17, 17. 80) | .234 | -0.02 (-12.20, 12.16) | .998 |
| LPA (mins/d) | 326.6 (49.9) | 318.0 (294.0, 361.6) | 333.1 (47.3) | 330.0 (294.1, 373.6) | 7.26 (1.07, 13.46) | .022 | 4.64 (-2.81, 12.09) | .223 |
| MVPA (mins/d) | 84.9 (26.0) | 80.6 (68.0, 99.3) | 84.1 (23.1) | 83.3 (66.7, 97.4) | -0.90 (-7.90, 6.10) | .800 | -4.71 (-11.82, 2.39) | .194 |

^a^ Interquartile range, presented as the interval from the 25^th^ to the 27^th^ percentile.

^b^ Mean differences between control and intervention groups from bootstrapped maximum likelihood linear mixed models with random intercepts for mothers groups. The model for the BMI z-score outcome was adjusted for baseline BMI z-score.

^c^ Adjustment for child age and sex, and maternal education. Additionally, models for BMI and waist circumference z-score outcomes were adjusted for mothers’ pre-pregnancy BMI reported at baseline, models for dietary outcomes were adjusted for child overall energy intake at that time point, and the physical activity outcome model was adjusted for average accelerometer wear time at that time point.

**eTable 2: Parent-reported service use and costs at follow-up (Costs in 2018 Australian dollars)**

|  | **First follow-up** | | **Second follow-up** | |
| --- | --- | --- | --- | --- |
|  | **Control** | **Intervention** | **Control** | **Intervention** |
| N reporting | 151 | 154 | 144 | 167 |
| Any service use | 42 (28.0%) | 43 (27.9%) | 19 (13.2%) | 23 (14.0%) |
| General Practitioner (doctor) | 24 (15.9%) | 24 (15.6%) | 6 (4.2%) | 13 (7.8%) |
| Maternal & Child Health Phone Helpline | 12 (7.9%) | 14 (9.1%) | 2 (1.4%) | 4 (2.4%) |
| Pediatrician | 13 (8.6%) | 14 (9.1%) | 4 (2.8%) | 5 (3.0%) |
| Dietitian | 9 (6.0%) | 4 (2.6%) | 6 (4.2%) | 7 (4.2%) |
| Complementary Practioner (e.g. Chiropractor, Osteopath, Naturopath) | 12 (7.9%) | 8 (5.2%) | 6 (4.2%) | 4 (2.4%) |
| Home visiting or outreach nurse | 2 (1.3%) | 4 (2.6%) | 1 (0.7%) | 1 (0.6%) |
| Mother-baby/parenting centre (day-stay) | 3 (2.0%) | 2 (1.3%) | 0 | 0 |
| Mother-baby/parenting centre (overnight) | 3 (2.0%) | 3 (1.9%) | 0 | 0 |
| Other provider (assorted) | 16 (10.7%) | 14 (9.1%) | 9 (6.3%) | 10 (6.0%) |
| Average cost per family to health sector: mean (SD)* | $193.32 ($697.18) | $123.66 ($450.93) | $42.30 ($181.75) | $18.38 ($96.77) |
| Average out-of-pocket cost: mean (SD) | $25.76 ($99.86) | $35.68 ($263.88) | $12.42 ($70.57) | $10.99 ($68.76) |

*Costs calculated based on following unit costs: General Practitioner $37.60 (Medical Benefits Scheme (MBS) Item 23); Maternal & Child Health Helpline $14.50 (based on 15min nurse time plus $2.50 overheads); Pediatrician $86.85 (MBS Item 104); Dietitian (MBS Item 10954); Complementary Practitioner $62.25 (MBS Items 10964 & 10966); Home visiting or outreach nurse (MBS Item 82210); Mother-baby/parenting centre day stay $388.15 (expert opinion); Mother-baby/parenting centre overnight $764.10 (expert opinion)
